# Biological membranes

**DOI:** 10.1042/bse0590043

**Published:** 2015-10-26

**Authors:** Helen Watson

**Affiliations:** University of Exeter Medical School, St. Luke's Campus, Magdalen Road, Exeter EX1 2LU, U.K.

## Abstract

Biological membranes allow life as we know it to exist. They form cells and enable separation between the inside and outside of an organism, controlling by means of their selective permeability which substances enter and leave. By allowing gradients of ions to be created across them, membranes also enable living organisms to generate energy. In addition, they control the flow of messages between cells by sending, receiving and processing information in the form of chemical and electrical signals. This essay summarizes the structure and function of membranes and the proteins within them, and describes their role in trafficking and transport, and their involvement in health and disease. Techniques for studying membranes are also discussed.

## Structure and organization of membranes

### Membranes are composed of lipids, proteins and sugars

Biological membranes consist of a double sheet (known as a bilayer) of lipid molecules. This structure is generally referred to as the phospholipid bilayer. In addition to the various types of lipids that occur in biological membranes, membrane proteins and sugars are also key components of the structure. Membrane proteins play a vital role in biological membranes, as they help to maintain the structural integrity, organization and flow of material through membranes. Sugars are found on one side of the bilayer only, and are attached by covalent bonds to some lipids and proteins.

**Figure 1.**
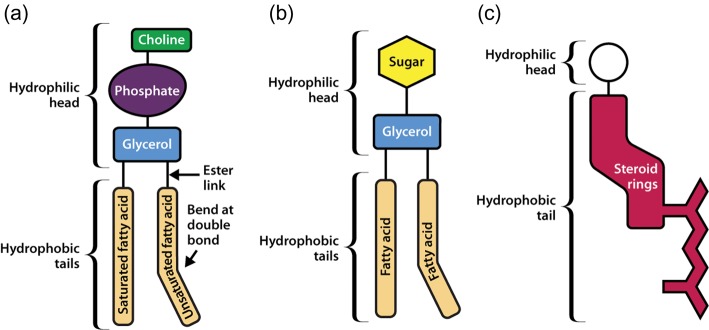
Schematic representations of three types of membrane lipid. (**a**) Phosphatidylcholine, a glycerophospholipid. (**b**) Glycolipid. (**c**) A sterol.

Three types of lipid are found in biological membranes, namely phospholipids, glycolipids and sterols. Phospholipids consist of two fatty acid chains linked to glycerol and a phosphate group. Phospholipids containing glycerol are referred to as glycerophospholipids. An example of a glycerophospholipid that is commonly found in biological membranes is phosphatidylcholine (PC) (Figure 1a), which has a choline molecule attached to the phosphate group. Serine and ethanolamine can replace the choline in this position, and these lipids are called phosphatidylserine (PS) and phosphatidylethanolamine (PE), respectively. Phospholipids can also be sphingophospholipids (based on sphingosine), such as sphingomyelin. Glycolipids can contain either glycerol or sphingosine, and always have a sugar such as glucose in place of the phosphate head found in phospholipids (Figure 1b). Sterols are absent from most bacterial membranes, but are an important component of animal (typically cholesterol) and plant (mainly stigmasterol) membranes. Cholesterol has a quite different structure to that of the phospholipids and glycolipids. It consists of a hydroxyl group (which is the hydrophilic ‘head’ region), a four-ring steroid structure and a short hydrocarbon side chain (Figure 1c).

The sugars attached to lipids and proteins can act as markers due to the structural diversity of sugar chains. For example, antigens composed of sugar chains on the surface of red blood cells determine an individual's blood group. These antigens are recognized by antibodies to cause an immune response, which is why matching blood groups must be used in blood transfusions. Other carbohydrate markers are present in disease (e.g. specific carbohydrates on the surface of cancer cells), and can be used by doctors and researchers to diagnose and treat various conditions.

### Amphipathic lipids form bilayers

**Figure 2.**
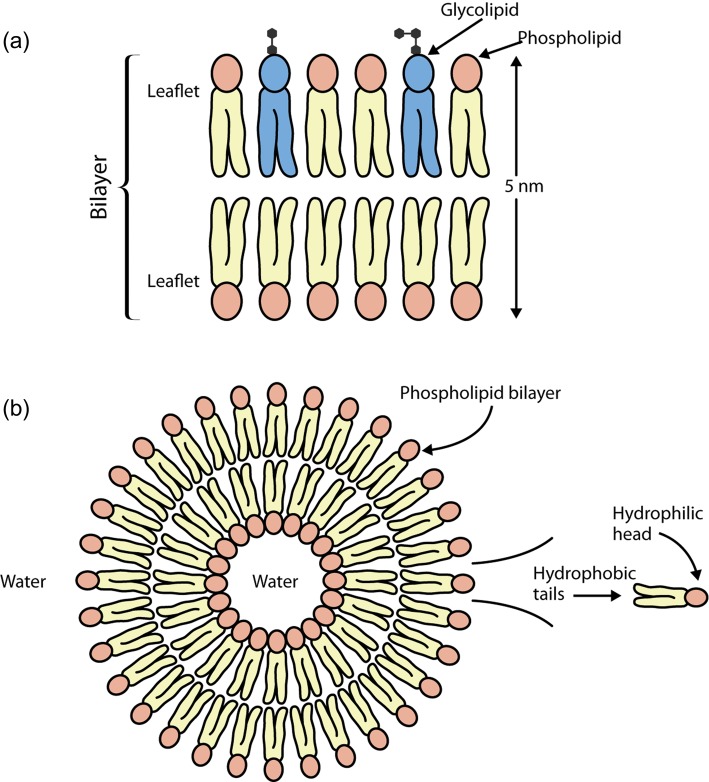
A membrane bilayer and liposome. Spontaneous formation of bilayers by membrane lipids. The hydrophilic heads (pink circles) will always face the aqueous environment in bilayers (**a**) and liposomes (**b**). The hydrophobic tails will face inward away from the water.

All membrane lipids are amphipathic—that is, they contain both a hydrophilic (water-loving) region and a hydrophobic (water-hating) region. Thus the most favourable environment for the hydrophilic head is an aqueous one, whereas the hydrophobic tail is more stable in a lipid environment. The amphipathic nature of membrane lipids means that they naturally form bilayers in which the hydrophilic heads point outward towards the aqueous environment and the hydrophobic tails point inward towards each other (Figure 2a). When placed in water, membrane lipids will spontaneously form liposomes, which are spheres formed of a bilayer with water inside and outside, resembling a tiny cell (Figure 2b). This is the most favourable configuration for these lipids, as it means that all of the hydrophilic heads are in contact with water and all of the hydrophobic tails are in a lipid environment.

Early experiments by E. Gorter and F. Grendel in 1925 were the first to demonstrate that biological membranes are bilayers. These researchers extracted the lipids from red blood cells and found that they occupied a space that was twice the surface area of the cell. Red blood cells contain no internal membranes, so they deduced that the plasma membrane must be composed of two layers of lipids.

### Biological membranes and the fluid mosaic model

The fluid mosaic model proposed by Jonathan Singer and Garth Nicolson in 1972 describes the dynamic and fluid nature of biological membranes. Lipids and proteins can diffuse laterally through the membrane. Phospholipids can diffuse relatively quickly in the leaflet of the bilayer in which they are located. A phospholipid can travel around the perimeter of a red blood cell in around 12 s, or move the length of a bacterial cell within 1 s. Phospholipids can also spin around on their head-to-tail axis, and their lipid tails are very flexible. These different types of movements create a dynamic, fluid membrane which surrounds cells and organelles. Membrane proteins can also move laterally in the bilayer, but their rates of movement vary and are generally slower than those of lipids. In some cases, membrane proteins are held in particular areas of the membrane in order to polarize the cell and enable different ends of the cell to have different functions. One example of this is the attachment of a glycosyl-phosphatidylinositol (GPI) anchor to proteins to target them to the apical membrane of epithelial cells and exclude them from the basolateral membrane.

**Figure 3.**
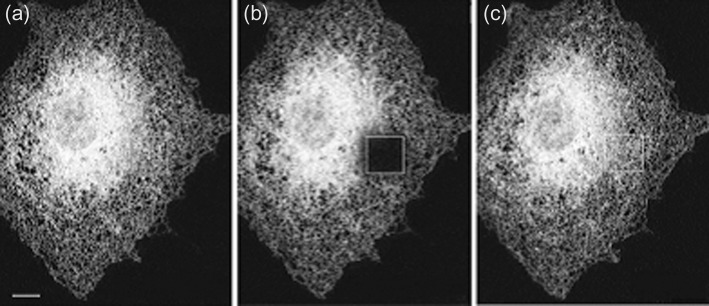
Photobleaching. Cells expressing a GFP-labelled protein in the endoplasmic reticulum were subjected to photobleaching. (**a**) A cell before bleaching. (**b**) The same cell immediately after bleaching of the square section shown. (**c**) The same cell 5 min after photobleaching. Adapted from Figure 1b from Lippincott-Schwartz, J., Snapp, E. and Kenworthy, A. (2001) Studying protein dynamics in living cells. Nat. Rev. Mol. Cell. Biol. **2**, 444–456.

Fluorescence photobleaching is one experimental method that is used by scientists to demonstrate visually the motility of proteins and lipids in a bilayer (Figure 3). A lipid or membrane protein located on the surface of a cell is tagged with a fluorescent marker such as green fluorescent protein (GFP). A beam of laser light is then focused on to a small area of the cell surface using a fluorescence microscope in order to bleach the fluorescent tags in this area so that they no longer emit a fluorescence signal. This small area of membrane is observed over time and gradually the fluorescence increases again, indicating that other tagged proteins or lipids are diffusing into this region from elsewhere in the membrane. This demonstrates that the lipid bilayer surrounding cells is fluid in nature and allows lateral diffusion of both lipids and membrane proteins.

Despite all this movement of lipids and proteins in the bilayer, vertical movement, or ‘flip-flop’, of lipids and proteins from one leaflet to another occurs at an extremely low rate. This is due to the energetic barrier encountered when forcing the hydrophilic head (in the case of lipids) or hydrophilic regions (in the case of proteins) through the hydrophobic environment of the inside of the membrane. This near absence of vertical movement allows the inner and outer leaflets of the bilayer to maintain different lipid compositions, and enables membrane proteins to be inserted in the correct orientation for them to function. However, some enzymes facilitate the process of lipid flip-flop from one leaflet to another. These flippases, or phospholipid translocators, use ATP to move lipids across the bilayer to the other leaflet. In eukaryotic cells, flippases are located in various organelles, including the endoplasmic reticulum (ER), where they flip-flop newly synthesized lipids.

### How membranes are made

Biological membranes are formed by adding to a pre-existing membrane. In prokaryotes this occurs on the inner leaflet of the plasma membrane, facing the cytoplasm. In eukaryotes, membrane synthesis takes place at the ER on the cytoplasmic leaflet of the ER membrane (termed the ‘inside’ of the cell). Lipids then leave the ER and travel through the secretory pathway for distribution to various subcellular compartments or the plasma membrane.

**Figure 4.**
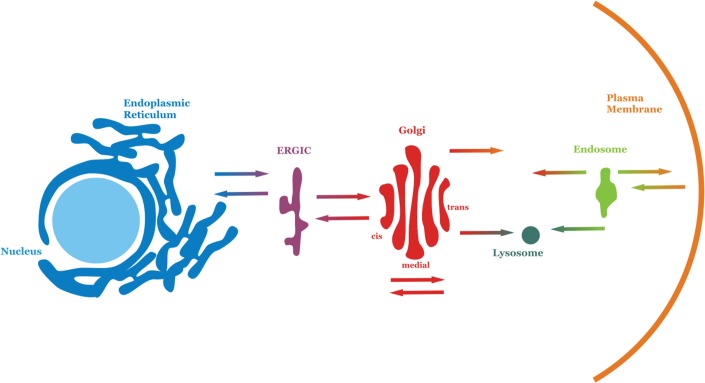
Membrane traffic in eukaryotic cells. The main compartments of eukaryotic cells are shown. Arrows indicate movement of lipid vesicles between them, with colours at the tail end indicating origin and those at the head end indicating destination.

In eukaryotic cells, enzymes that span the ER catalyse the formation of membrane lipids. In the cytoplasmic leaflet of the ER membrane, two fatty acids are bound, one by one, to glycerol phosphate from the cytoplasm. This newly formed diacylglycerol phosphate is anchored in the ER membrane by its fatty acid chains. The phosphate is then replaced by the head group (e.g. phosphate and choline). Flippases in the ER membrane can then move some of these newly formed lipids to the luminal side of the ER membrane. Similarly, flippases in prokaryotes can transfer new lipids from the inner leaflet of the plasma membrane to the outer leaflet. These flippases are responsible for adjusting the lipid composition of each layer of the membrane. In eukaryotes, lipids must then be distributed to the various intracellular membranes. The traffic of vesicles between organelles in combination with signals that direct particular lipids to specific locations is required to create the correct lipid composition in all of the cellular membranes (Figure 4). Vesicles bud from the ER and travel via the ER–Golgi intermediate compartment (ERGIC) to join with the Golgi, where sorting of lipids takes place. The Golgi then sends lipids in vesicles to various destinations, including the plasma membrane and lysosomes. Lipids and proteins are internalized from the plasma membrane into endosomes. Organelles, such as mitochondria, acquire lipids from the ER by a different mechanism. Water-soluble proteins called phospholipid-exchange proteins remove phospholipids from the ER membrane and deposit them in the membranes of the appropriate organelles.

### Distribution of lipids

The inner and outer leaflets of bilayers differ in their lipid composition. In mammalian cells, the outer leaflet of the plasma membrane contains predominantly PC and sphingomyelin, whereas PS and PE are found on the inner leaflet. During programmed cell death (apoptosis), PS is no longer restricted to the inner leaflet of the plasma membrane. It is exposed on the outer leaflet by the action of an enzyme called scramblase which is a type of flippase enzyme. PS is negatively charged, unlike PC, which has no net charge. The movement of PS into the outer leaflet therefore changes the charge of the plasma membrane as viewed from the outside of the cell. This change in surface charge labels the apoptotic cell for phagocytosis by phagocytic cells such as macrophages.

Lipid composition also varies between the organelles within eukaryotic cells. Cholesterol is synthesized in the ER, but the ER membrane has a relatively low cholesterol content, as much of the cholesterol is transported to other cellular membranes. The prevalence of cholesterol in membranes increases through the secretory pathway, with more in the Golgi than in the ER (the *trans*-Golgi network is richer in cholesterol than the *cis*-Golgi), and most in the plasma membrane. This increase in cholesterol through the secretory pathway results in slightly thicker membranes in the late Golgi and plasma membrane compared with the ER, and is thought to be a contributing factor to protein sorting through the pathway, as membrane proteins in the plasma membrane generally have longer hydrophobic transmembrane domains than membrane proteins that reside in the ER.

## Membrane proteins

Membrane proteins are the nanomachines that enable membranes to send and receive messages and to transport molecules into and out of cells and compartments. Without membrane proteins the phospholipid membrane would present an impenetrable barrier and cells would be unable to communicate with their neighbours, transport nutrients into the cell or waste products out of it, or respond to external stimuli. Both unicellular and multicellular organisms need membrane proteins in order to live. The membrane proteins that are present in a particular membrane determine the substances to which it will be permeable and what signal molecules it can recognize.

### Synthesis of membrane proteins

**Figure 5.**
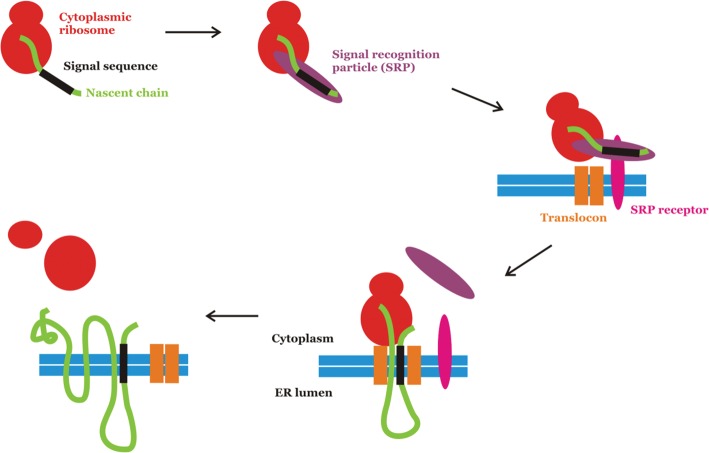
Co-translational ER protein targeting. The key steps of ER targeting are summarized. Each component is labelled and the ER membrane is represented by double blue lines. The signal sequence (shown in black) becomes the first transmembrane domain of the protein in this example.

In eukaryotic cells, the synthesis of membrane proteins destined for the plasma membrane, ER or any other membrane-bound compartment begins on cytosolic ribosomes. After a short segment of protein has been synthesized, the ribosome, mRNA and nascent protein chain associate with the ER, where the rest of the protein is made and simultaneously inserted into the membrane. This phenomenon was first explained by Günter Blobel, David Sabatini and Bernhard Dobberstein in the 1970s. These scientists proposed that there is a ‘binding factor’ which recognizes the emerging protein chain and can dock the ribosome at the ER membrane. We now know that there is an N-terminal signal sequence within membrane proteins. These signal sequences are not identical but share a common motif, namely a hydrophobic stretch of 20–30 amino acids, a basic region at the N-terminus and a polar domain at the C-terminus of the signal. These N-terminal signal sequences are recognized by the signal recognition particle (SRP), which has binding sites for the signal sequence, ribosome and the SRP receptor which is embedded in the ER membrane. Upon binding the SRP, the ribosome pauses protein synthesis. The SRP binds to the SRP receptor, adjacent to a translocon pore in the ER membrane. The translocon is a protein pore through which membrane protein chains can be threaded into the membrane. It has a laterally opening gate to allow newly synthesized proteins into the ER membrane. Once the ribosome is at the translocon, the SRP dissociates and protein synthesis resumes. This process is referred to as co-translational targeting, and the main events are summarized in Figure 5.

Co-translational targeting is the dominant mechanism for protein delivery to the ER in higher eukaryotes, whereas yeast and prokaryotes favour post-translational targeting, whereby proteins are delivered to the ER after completion of synthesis. Post-translational targeting also occurs in higher eukaryotes, often when a membrane protein is so small that the signal sequence does not emerge until the whole protein has been synthesized. Post-translational targeting can be carried out both by SRP-dependent and by SRP-independent mechanisms.

### Structure and function of membrane proteins

Membrane-spanning proteins are diverse in structure and function. They can be constructed of α-helices or from β-barrels. The β-barrel membrane proteins often function as pores, with hydrophobic amino acids facing out into the bilayer. In addition, there are other non-spanning proteins which associate with the bilayer, often using a hydrophobic anchor. Here we shall focus on the α-helical membrane proteins. These proteins have at least one α-helical hydrophobic stretch of amino acids, around 20 residues in length, which corresponds to around 30 Å (the thickness of an average phospholipid bilayer). If an α-helical membrane protein spans the membrane more than once, it will have more than one of these hydrophobic sections. For example, the Ca^2+^-ATPase of the ER and sarcoplasmic reticulum (SR) spans the membrane 10 times, so it has 10 hydrophobic stretches of around 20 amino acids each.

### Membrane proteins control what enters and leaves the cell

A vital class of membrane proteins are those involved in active or passive transport of materials across the cell membrane or other subcellular membranes surrounding organelles. For a cell or an organism to survive, it is crucial that the right substances enter cells (e.g. nutrients) and the right substances are transported out of them (e.g. toxins).

#### Passive and active transport

**Figure 6.**
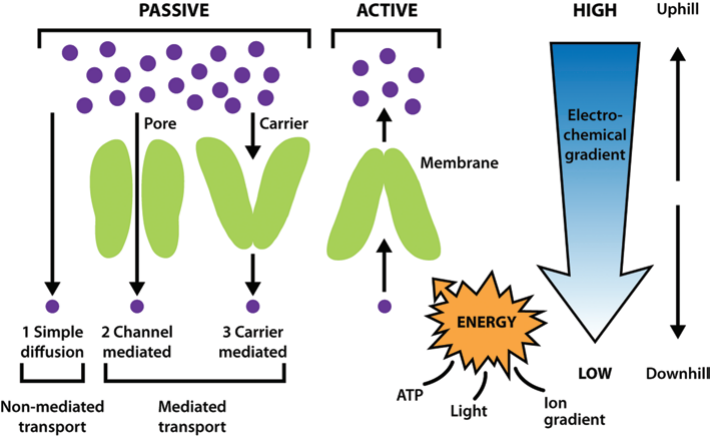
Passive and active transport. The different types of membrane proteins involved in passive and active transport are shown.

Molecules can cross biological membranes in several different ways depending on their concentration on either side of the membrane, their size and their charge. Some molecules, including water, can simply diffuse through the membrane without assistance. However, large molecules or charged molecules cannot cross membranes by simple diffusion. Charged molecules such as ions can move through channels passively, down electrochemical gradients. This movement is described as ‘downhill’, as the ions or molecules travel from an area of high concentration to an area of low concentration. This requires channel proteins but no energy input. Passive transport can also be mediated by carrier proteins that carry specific molecules such as amino acids down concentration gradients, again without any requirement for energy. Active transport moves species against concentration gradients and requires energy, which is obtained from ATP, from light, or from the downhill movement of a second type of molecule or ion within the same transporter (Figure 6).

##### Passive transport

Passive transport is the movement of molecules across biological membranes down concentration gradients. This type of transport does not require energy. Channels form water-filled pores and thus create a hydrophilic path that enables ions to travel through the hydrophobic membrane. These channels allow downhill movement of ions, down an electrochemical gradient. Both the size and charge of the channel pore determine its selectivity. Different channels have pores of different diameters to allow the selection of ions on the basis of size. The amino acids that line the pore will be hydrophilic, and their charge will determine whether positive or negative ions travel through it. For example, Ca^2+^ is positively charged, so the amino acids lining the pores of Ca^2+^ channels are generally basic (i.e. they carry a negative charge).

**Figure 7.**
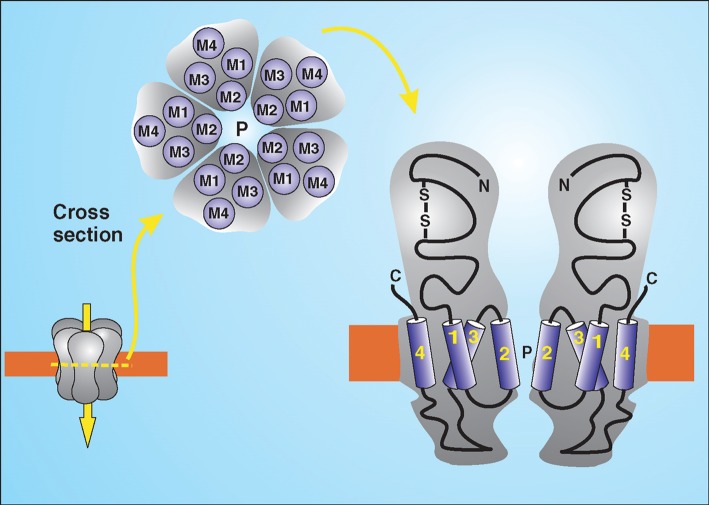
The nicotinic acetylcholine receptor. The pentameric structure of the receptor is shown, with the pore region (P) indicated. Transmembrane helices (M1–M4) are labelled in each subunit. The bilayer is shown in orange. Reproduced from Berridge, M.J. (2012) Cell Signalling Biology; doi:10.1042/csb0001003, with permission.

Channels are not always open. They can be gated by ligands which bind to some part of the protein, either by a change in membrane potential (voltage gated) or by mechanical stress (mechanosensitive). The nicotinic acetylcholine receptor is an example of a ligand-gated ion channel which opens upon binding the neurotransmitter acetylcholine (Figure 7). The nicotinic acetylcholine receptor is a pentameric membrane protein composed of five subunits arranged in a ring, with a pore through the centre. In the closed state, the pore is blocked by large hydrophobic amino acid side chains which rotate out of the way upon acetylcholine binding to make way for smaller hydrophilic side chains, allowing the passage of ions through the pore. Opening of the nicotinic acetylcholine receptor allows rapid movement of Na^+^ ions into the cell and slower movement of K^+^ ions out of the cell, in both cases down the electrochemical gradient of the ion. The difference in gradients between Na^+^ and K^+^ across the membrane means that more Na^+^ enters the cell than K^+^ leaves it. This creates a net movement of positive charges into the cell, resulting in a change in membrane potential. Acetylcholine released by motor neurons at the neuromuscular junction travels across the synapse and binds to nicotinic acetylcholine receptors in the plasma membrane of the muscle cells, causing membrane depolarization. This depolarization of the muscle cells triggers Ca^2+^ release and muscle contraction.

Carrier proteins are the other class of membrane proteins, apart from channels, which can facilitate passive transport of substances down concentration gradients. Carrier proteins transport molecules much more slowly than channels, as a number of conformational changes in the carrier are required for the transport of the solute across the membrane. A molecule such as a sugar binds to the carrier protein on one side of the membrane where it is present at a high concentration. Upon binding, the carrier changes conformation so that the sugar molecule then faces towards the opposite side of the membrane. The concentration of sugar on this side is lower, so dissociation occurs and the sugar is released. This is downhill movement and, although slower than movement through channels, it requires no energy.

The cystic fibrosis transmembrane conductance regulator (CFTR) is an ATP-dependent chloride ion (Cl^−^) channel that has an important role in regulating the viscosity of mucus on the outside of epithelial cells. ATP is used to gate the channel, but the movement of Cl^−^ occurs down its electrochemical gradient, so does not require energy. A heritable change in the *CFTR* gene which results in a single amino acid deletion in the protein causes cystic fibrosis. This is a serious illness in which thick mucus accumulates in the lungs, causing a significantly lower than average life expectancy in patients who have the disease. Unimpaired ion transport is vital for our survival and health, and conditions such as cystic fibrosis highlight the need for research into these types of proteins.

##### Active transport

The transport of molecules across a membrane against a concentration gradient requires energy, and is referred to as active transport. This energy can be obtained from ATP hydrolysis (primary active transport), from light (as, for example, in the case of the bacterial proton pump bacteriorhodopsin), or from an electrochemical gradient of an ion such as Na^+^ or H^+^ (secondary active transport).

**Figure 8.**
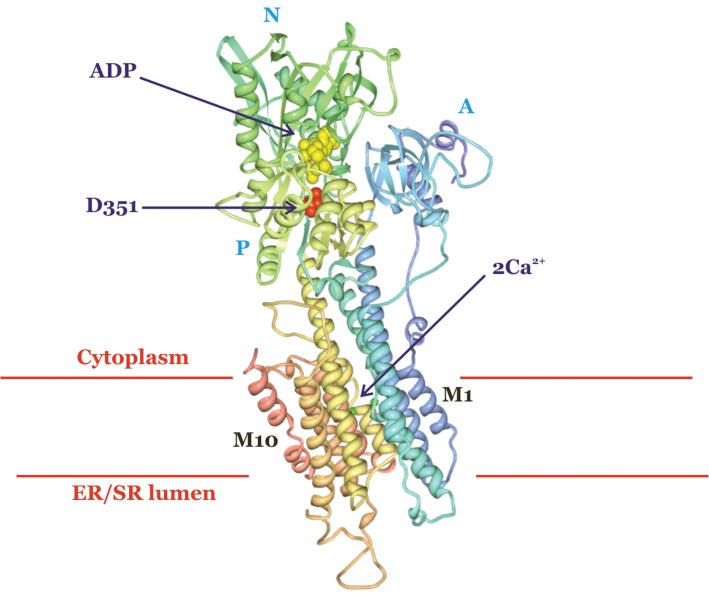
The sarco/endoplasmic reticulum Ca^2+^-ATPase (SERCA). The crystal structure of SERCA in the ADP- and Ca^2+^-bound state is shown. D351 (in red) is the residue phosphorylated during the movement of Ca^2+^ ions into the ER or SR. The three cytoplasmic domains, phosphorylation (P), nucleotide binding (N) and actuator (A) are labelled. ADP is shown in yellow and Ca^2+^ ions in green. Protein Data Bank (PDB) code 2ZBD, rendered using PDB Protein Workshop.

Calcium ions signal many events, including muscle contraction, neurotransmitter release and cellular motility. However, high cytoplasmic concentrations of Ca^2+^ are toxic to the cell. Therefore Ca^2+^ must be tightly regulated and removed from the cytoplasm either into internal stores (the ER, and the SR in muscle cells) or into the extracellular space. This Ca^2+^ removal is carried out by a family of Ca^2+^-ATPases, including the sarco/endoplasmic reticulum Ca^2+^-ATPase (SERCA), which hydrolyse ATP to move Ca^2+^ against its electrochemical gradient into the ER and SR (Figure 8). There are Ca^2+^-ATPases in the ER, Golgi and plasma membrane, and despite their sequence similarity, these proteins are differentially targeted to the appropriate membrane. These Ca^2+^ pumps are primary active transporters. SERCA moves two Ca^2+^ ions into the ER or SR for every ATP molecule that is hydrolysed. The pump undergoes a cycle of binding ATP and phosphorylation, and undergoes large conformational changes every time it transports a pair of Ca^2+^ ions. SERCA is a P-type ATPase (so called because it is phosphorylated during ion transport). There are many P-type ATPases, and they are conserved in evolution across many species. The Na^+^/K^+^-ATPase is one of these P-type ATPases, and it works in a similar way to SERCA to pump Na^+^ out of the cell and K^+^ into the cell using energy derived from the hydrolysis of ATP. We have now obtained three-dimensional structures of SERCA in a number of conformational states, which allow scientists to visualize the transport process.

**Figure 9.**
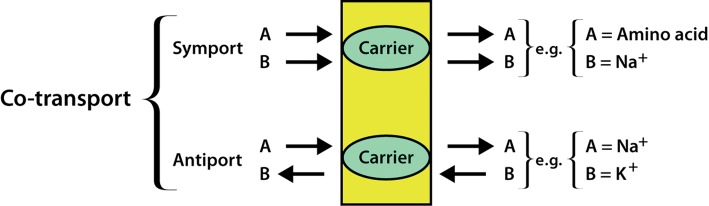
Symport and antiport. The two types of co-transport are shown, with examples.

Secondary active transport requires an ion electrochemical gradient to drive the uphill transport of another solute. The downhill movement of one species drives the uphill movement of the other. This can be symport (in which both types of molecule or ion travel across the membrane in the same direction) or antiport (in which the two species travel in opposite directions), as shown in Figure 9.

In order to transport glucose into cells, the Na^+^–glucose symporter uses the electrochemical gradient of Na^+^ across the plasma membrane. The concentration of Na^+^ is much higher outside the cell, and the inside of the cell is negatively charged relative to the outside, so by allowing Na^+^ to travel down its electrochemical gradient, these transporters can move glucose uphill, into the cell and against its concentration gradient. This is referred to as symport, as both Na^+^ and glucose travel in the same direction—in this case into the cell. In order for this symport to be sustainable, the Na^+^ gradient must be maintained. This is done by the Na^+^/K^+^-ATPase, which uses ATP to pump the Na^+^ back into the extracellular space, thus maintaining a low intracellular Na^+^ concentration.

Both Na^+^ and Ca^2+^ are present at much higher concentrations outside the cell than inside it. Like the Na^+^–glucose symporter, the Na^+^–Ca^2+^ exchanger uses the electrochemical gradient of Na^+^ across the plasma membrane to move a second species (Ca^2+^) against its electrochemical gradient. However, in this case the transporter is an antiporter, as it uses the concentration gradient of one substance moving in (Na^+^) to move another (Ca^2+^) out of the cell. This antiporter has an exchange rate of three Na^+^ ions in to two Ca^2+^ ions out. It moves Ca^2+^ out of the cell faster than the plasma membrane equivalents of SERCA, but has a lower affinity for Ca^2+^ than these P-type ATPases. Again this transporter relies on the Na^+^/K^+^-ATPase to maintain the low intracellular Na^+^ concentration.

### Solving the structure of membrane proteins

In order to understand more fully the mechanisms of action of membrane proteins such as the transporters described here, we can determine their three-dimensional protein structures. As a result of huge advances in structural biology in the last 50 years, we now have access to many thousands of protein structures in online databases. This enables researchers to visualize the structure of their protein of interest, and thus gain insight into its mechanism.

#### X-ray crystallography

The structure of whale myoglobin was solved in 1958 using X-ray crystallography, earning John C. Kendrew and Max Perutz the Nobel Prize in Chemistry. This was the first protein structure to be solved using this technique, and since then thousands of proteins have been solved using this method. X-ray crystallography works by firing a beam of X-rays at a crystalline structure and measuring the diffraction of the X-rays after they have passed through the structure of interest. This generates an electron density map, showing where different atoms in the structure are located. For regular crystalline solids such as salts this is relatively straightforward, but for large irregular molecules such as proteins it can present many technical challenges. Before a protein is subjected to X-ray beams, it must first be purified and crystallized. In nature, proteins exist in the busy milieu of a cell, surrounded by thousands of other types of proteins, as well as lipids and other molecules. A common method of obtaining enough of the protein of interest involves expressing the relevant gene in a system such as bacteria. The gene is tagged with a small protein tag which can be used to isolate the protein of interest. Bacterial systems allow large amounts of protein to be produced cheaply and quickly. However, if the protein of interest is from a species that is only distantly related to that in which it is normally expressed (e.g. a human protein produced in *Escherichia coli* (*E. coli*)), the lack of correct glycosylation enzymes and the differences in protein folding and assembly may prevent the production of a biologically active protein. In addition, the expression of membrane proteins that make pores or channels can kill the host organism.

A pure protein sample is then crystallized by allowing water to evaporate away, in exactly the same way as a solution of salt will form crystals naturally when left to dry. Optimum conditions for this must be determined, and crystallization conditions are not always straightforward, as they differ from one protein to another. For soluble proteins such as myoglobin this is easier than for insoluble membrane proteins. Membrane proteins have lipid-soluble domains that will not dissolve in an aqueous medium. This significantly decreases the ease with which membrane protein structures can be solved using X-ray diffraction. However, there are ways in which scientists can overcome this difficulty. Generally, membrane proteins are removed from the membrane in which they were made and placed in an environment of lipids and detergents for crystallization. Sometimes the lipids associated with the protein are apparent in the crystal structure.

**Figure 10.**
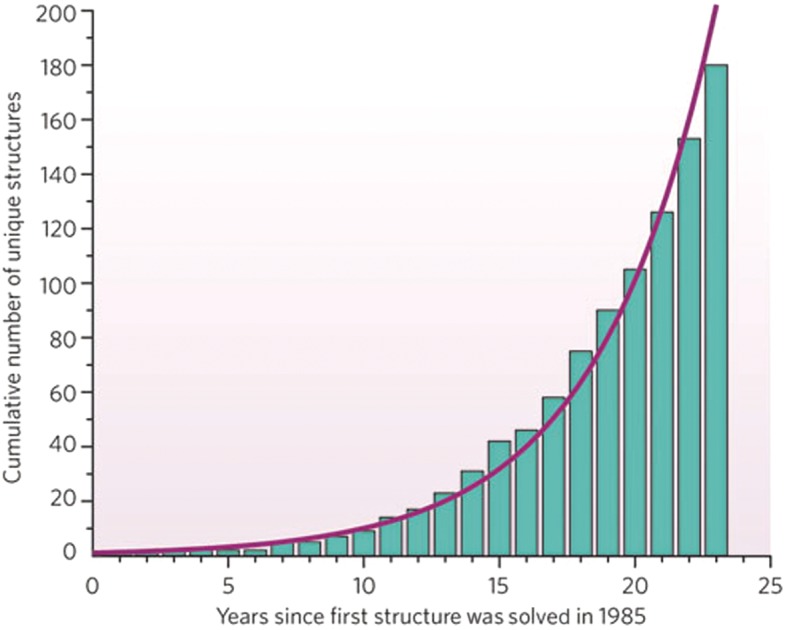
X-ray crystal structures of membrane proteins. The increase in the number of solved crystal structures of membrane proteins is shown from 1985, when the first such structure was solved. Adapted from White, S.H. (2009) Biophysical dissection of membrane proteins. Nature **459**, 344–346.

The number of solved crystal structures of proteins is constantly growing as technology improves and expertise is shared among scientists to help to optimize conditions for crystal production. The Protein Data Bank (PDB) is an online archive of protein structures which can be freely accessed by scientists worldwide. At the time of writing, 88% of the structures in the PDB have been solved by X-ray crystallography, and there are currently just under 70 000 X-ray crystal structures in the database. The number of membrane protein structures in the PDB is increasing rapidly with the refinement of crystallization techniques (Figure 10).

#### Other structural techniques

Nuclear magnetic resonance (NMR) spectroscopy is another valuable technique for elucidating membrane protein structure. Molecules are placed in a magnetic field and the resonance properties of different atomic nuclei are measured, which gives an indication of where in a particular molecule different atoms are located. Generally, NMR is limited to smaller proteins, below around 35 kDa in size. It also offers the potential to visualize proteins in a more physiologically relevant environment (e.g. in lipid bilayers or micelles). Another advantage of NMR is that it does not require the protein to be locked in a crystal lattice—a structure which can distort the natural shape of the protein.

Electron microscopy can also be used to study membrane protein structure. By freezing membrane proteins in their natural lipid environments, it is possible to investigate their structure using high-resolution electron microscopy. This provides a snapshot of the naturally occurring conformation of individual proteins in the bilayer.

### Interactions between lipids and proteins in biological membranes

The lipids that surround membrane proteins in biological membranes play an important role in the activity of these proteins. As was mentioned earlier, some membrane protein crystal structures include lipids bound to the outside surface of the transmembrane domains of the proteins. It is thought that these lipids bind tightly to the protein, and have a long-lived interaction with the transmembrane region. In other cases, lipids are thought to interact briefly with membrane proteins, rapidly moving away and being replaced by other membrane lipids. The activity of membrane proteins is considered to be dependent to some extent on the lipids that surround them in the membrane. Certain types of K^+^ channel are thought to bind to negatively charged membrane lipids, as the activity of these channels increases at higher anionic lipid concentrations. These types of interaction can be studied by placing a purified form of the protein of interest in an artificial bilayer and measuring its activity. By altering the types of lipid present in the artificial bilayer, deductions can be made about the lipids that the protein requires in order to be active. Fluorescence spectroscopy and electron spin resonance are two techniques that are used to measure how strongly membrane proteins interact with specific lipids around them.

Molecular dynamics simulations use computer algorithms to work through theoretical problems. These simulated experiments are useful for investigating interactions between membrane proteins and lipids, as in real membranes these interactions are often so fleeting that they are very difficult to measure. Molecular dynamics simulations have predicted that in the case of the nicotinic acetylcholine receptor, the negatively charged lipid, phosphatidic acid, is required for activity. These simulations have also shown that cholesterol stabilizes the receptor and that the phosphatidic acid forms a shell around the protein which is more long-lasting than the interactions with other membrane lipids. Although molecular dynamics simulations are extremely useful, they are limited by the assumptions and approximations on which they are based. As in many areas of biology, a combination of experimental and computational research is required if real progress is to be made in understanding the complexity of biological membranes.

## Internal membranes in eukaryotic cells form organelles

Inside the plasma membrane that surrounds eukaryotic cells lie many other membranes which define the intracellular compartments, or organelles. Each of these organelles has distinct functions and contains specific complements of proteins adapted for these roles. With the exception of a few proteins that are coded for by the mitochondrial genome, synthesis of all of the proteins that are required in these organelles begins on ribosomes in the cytoplasm, and therefore the proteins must be directed to the correct destination. We have seen earlier how this is achieved with membrane proteins, and most organelles have some kind of signal sequence that can be recognized by various receptors and which ensures that the protein arrives at the correct organelle.

### Organelles have distinct lipid compositions

Besides the specific protein complement of each organelle, the lipid make-up of the bilayers surrounding organelles varies. Lipids are synthesized in the ER, and flippases move lipid molecules between leaflets of the bilayer. For organelles in the secretory pathway and the plasma membrane, lipid transport into these compartments is mediated by vesicular membrane traffic through the pathway. The cholesterol concentration in membranes increases from the ER through the Golgi to the plasma membrane. Cholesterol makes membranes thicker and more rigid, so the low levels of cholesterol in the ER membrane render it thin and facilitate the insertion of newly synthesized membrane and secretory proteins. PC becomes relatively less abundant through this pathway, with more found in the ER than at the plasma membrane. PS and PE are found throughout the secretory pathway in the cytosolic leaflet of the membranes. This differential lipid composition through the secretory pathway is achieved by targeting specific lipids into transport vesicles. Proteins included in these vesicles act as labels and direct the lipids to the right compartment. Forward-moving (anterograde) vesicles destined for the plasma membrane are rich in cholesterol. Lipids also move backwards through the secretory pathway, from the plasma membrane towards the ER. This is known as retrograde traffic. Retrograde vesicles from the Golgi are enriched in lipids such as PC, which are concentrated in the ER.

The lipid composition of the mitochondria is very different from that of the secretory pathway compartments. Mitochondrial membranes are much richer in PE and cardiolipin than is the ER. Cardiolipin is synthesized in the mitochondria and is predominantly confined to this organelle. As membrane proteins have evolved along with their organelles and surrounding lipids, it follows that different lipid compositions are required in different organelles for the optimum activity of the proteins within their membranes. The structure of the ADP/ATP carrier in mitochondria has been solved and was found to include cardiolipin and PC molecules bound to the protein. The activity of this carrier protein is dependent on the presence of cardiolipin, which is relatively abundant in mitochondrial membranes.

### Proteins must be targeted to the correct organelle for cells to function

The targeting of newly synthesized membrane and secretory proteins to the ER has already been briefly discussed. However, there are many different destinations within the cell to which a protein can be sent, and sometimes proteins are located in more than one of these. The signals and protein machinery that are required to target proteins to the correct compartment are many and various, and much of the detail of the exact mechanisms involved has yet to be clarified.

### Vesicular transport

**Figure 11.**
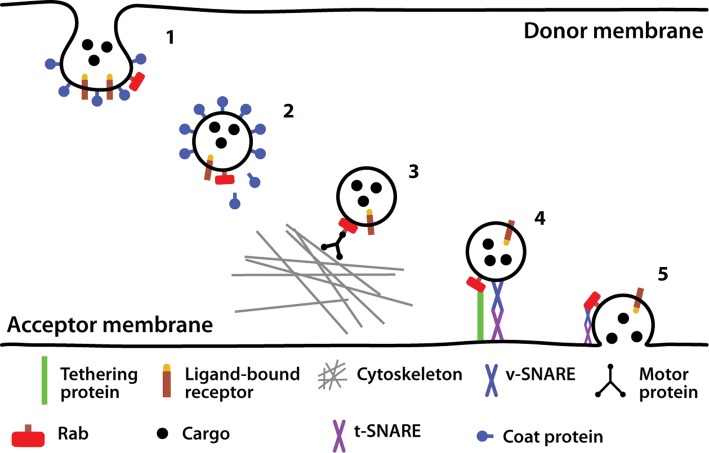
Vesicle production, transport and fusion. The main events in vesicular transport of cargo are shown. Cargo is selected and packed into vesicles which are formed by coat proteins (1 and 2). The GTPase Rab is also incorporated on the outside of the vesicle and facilitates the steps illustrated. The vesicle then travels along proteins of the cytoskeleton towards its destination following dissociation of coat proteins (3). The vesicle is tethered to the donor membrane with the help of tethering proteins and the SNARE complexes (4), allowing membrane fusion and release of the cargo (5).

Traffic through the secretory pathway is by vesicular transport in both anterograde and retrograde directions. Proteins and lipids can be included and excluded from vesicles by various means in order to selectively determine which molecules move forward or backward through the pathway. Vesicles are coated with proteins that determine their destination. Generally these coat proteins (COPs) are directional—COPII coats anterograde vesicles, and COPI coats retrograde vesicles. Proteins that travel in vesicles (referred to as cargo) are selected either by interacting with receptors in the vesicles or by directly interacting with the coat proteins. The selection of cargo occurs at the budding stage, when the coat proteins begin to distort the donor membrane (e.g. the ER) into a vesicle. Once the cargo has been selected and the coat proteins have been assembled, the vesicle buds off and travels to the acceptor membrane (e.g. the Golgi in the case of COPII vesicles from the ER) either by diffusion or with the help of motor proteins that ‘walk’ the vesicle along the cytoskeleton. The vesicle then fuses with the acceptor membrane, depositing its cargo and constituent lipids (Figure 11).

The formation and fusing of vesicles are energetically demanding, as these processes require the stable bilayer to be broken in order to pinch off a new vesicle, and then fused with a different membrane. Both energy and specialized protein machinery are required to overcome this energy barrier.

Once the vesicle has budded, travelled through the cytosol and reached its destination, it must fuse with its receptor membrane. Again this is an energetically unfavourable process, and protein machinery has to be utilized in order to allow the fusion of two bilayers. SNARE proteins are central to the process of vesicle fusion. Vesicles carry v-SNAREs that bind to specific t-SNAREs on the target membranes. Not only does this confer specificity in the targeting of vesicles, but also the SNAREs facilitate membrane fusion on arrival of the vesicle. The interacting v-SNAREs and t-SNAREs form a four-helix bundle at the interface between the two membranes, consisting of three helices from the t-SNARE and one helix from the v-SNARE. This stable interaction is thought to provide the free energy necessary to enable the two membranes to become very close and fuse. As the two bilayers become closer, the lipids in the two outer leaflets can come into contact with one another, thereby increasing the hydrophobic nature of the site and enabling the membranes to join, and overcoming the energy barrier. The transmembrane domains of the SNAREs are also believed to be involved in membrane fusion, as when they are replaced by lipids experimentally, fusion does not occur. Upon fusion, the cargo enters the target compartment, and the lipids and membrane proteins that formed the vesicle diffuse into the target membrane.

Determining the mechanisms of membrane budding is important for understanding how viruses such as the human immunodeficiency virus (HIV) produce new viral particles. Unlike the budding in the secretory pathway described earlier, HIV particles bud away from the cytoplasm, into the extracellular space. This viral budding occurs in the same orientation as the budding that occurs within endosomes. The proteins which enable this budding are referred to as endosomal sorting complexes required for transport (ESCRTs). HIV ‘hijacks’ the ESCRT machinery to enable it to bud from the plasma membrane, out of the cytoplasm and into the extracellular space. Interactions between HIV proteins and ESCRT proteins recruit the host cell ESCRT machinery to the budding vesicle, allowing membrane scission and vesicle release. Other viruses can bud without assistance from the ESCRTs, and it is thought that HIV may also be able to bud in an ESCRT-independent manner. Understanding more about these membrane budding and scission events is crucial to elucidating how viruses proliferate and how we can inhibit processes by means of drug interventions.

### Protein trafficking in the secretory pathway

As described earlier, a hydrophobic stretch of 20–30 amino acids with a basic N-terminus and a polar region at the C-terminus emerging from the ribosome causes the protein to be targeted to the ER, where synthesis is completed. This hydrophobic stretch can be cleaved in the case of soluble proteins, or it can remain attached. An uncleaved signal sequence is referred to as a signal anchor sequence, as it both signals ER targeting and then goes on to anchor a protein in the membrane, and becomes a transmembrane domain in the fully folded protein. The SRP-dependent targeting step is common to ER proteins as well as proteins destined for the Golgi or the plasma membrane, or to be secreted from the cell.

ER exit is thought to allow some selection of which proteins remain in the ER and which proteins leave and move in vesicles towards the Golgi. ER exit sites are located in areas of the ER close to the Golgi, and are rich in COPII coat proteins. It is not understood exactly which properties of a protein determine whether it will leave the ER in COPII vesicles, but it is currently thought that the transmembrane domain length is an important factor. Longer transmembrane domains appear to predispose proteins to exit the ER and travel towards the Golgi. This is consistent with the fact that membrane thickness increases through the secretory pathway due to increased cholesterol content, as described earlier.

Upon arrival at the *cis*-Golgi, proteins can then be retrieved to the ER, remain in the Golgi, or travel onward to the plasma membrane. Retrieval to the ER is not fully understood, but some proteins contain retrieval motifs, such as the KDEL four-amino-acid motif which is recognized by a receptor and enables packaging of the protein into retrograde COPI vesicles. Other proteins appear to cycle between the ER and the Golgi without known retrieval motifs. Proteins move in both anterograde and retrograde directions through the Golgi stack. They can then leave the *trans*-Golgi and move to the plasma membrane in vesicles.

Proteins at the plasma membrane can move into the cell in vesicles by endocytosis (e.g. when surface receptors are internalized for degradation in lysosomes). Endocytic vesicles are often clathrin coated. Clathrin, like the COPs, distorts the membrane into curved structures, allowing vesicle formation. Clathrin forms a cage-like shape that promotes vesicle formation and scission by virtue of the rigid shape of the protein complexes which form at the membrane. Not all endocytosis is clathrin dependent, and there are other proteins, such as caveolin, which can facilitate the formation of endocytic vesicles.

### Mitochondrial and nuclear protein targeting

Newly synthesized proteins destined for the mitochondria or the nucleus are targeted in a different way, independently of the secretory pathway. Some mitochondrial proteins are encoded by the mitochondrial genome, while others are encoded by the nuclear genome. Mitochondria have a double-layered membrane. Therefore targeting signals for mitochondrial proteins need to contain information not only to direct the protein to the organelle, but also to determine in which membrane it will be located (in the case of membrane proteins), or whether it will be located inside the mitochondria (the matrix) or in the intermembrane space between the inner and outer membranes (in the case of soluble proteins). Mitochondrial targeting motifs vary enormously, but generally are located at the N-terminus of the protein and are rich in positively charged and hydrophobic amino acids. Proteins destined for the nucleus are targeted by nuclear localization sequences that direct proteins which have been synthesized in the cytoplasm through nuclear pore complexes. Again these sequences are not very highly conserved, but generally contain clusters of positively charged amino acids.

## Sending messages across membranes

We have already seen how ion channels and other transport proteins can allow substances to cross the lipid bilayer. Knowledge of how fat-soluble and water-soluble substances cross membranes is important for gaining an understanding of how messages cross membranes and thus how one cell can communicate with another. Cells receive and send messages constantly (e.g. in order to respond to hormone signals, conduct action potentials, and sense external stimuli such as taste and smell).

### Messengers: lipid soluble or water soluble?

Substances that send messages are known as messengers, and they vary enormously in their chemical composition, size and hydrophobicity. In order to understand how a cell receives a message, it is important to ascertain first whether the messenger is lipid or water soluble. Hormones are one example of messengers that are released by cells. The human body contains both lipid-soluble and water-soluble hormones. Lipid-soluble hormones are generally transported through the blood, bound to carrier proteins. Steroid hormones such as the androgens and oestrogens are lipid soluble by virtue of their ringed molecular structures, which are derived from cholesterol. This allows these hormones to diffuse freely through the plasma membrane of cells and bind to their receptors, which are located inside cells. In the case of oestrogen, the receptor is located in the cytoplasm and upon ligand binding relocates to the nucleus, where it binds DNA and acts as a transcription factor, altering gene expression. The receptor contains a nuclear localization sequence which is hidden until oestrogen binds, allowing it to be targeted to the nucleus.

Other hormones, such as insulin and adrenaline, are water soluble and therefore cannot pass freely through the membranes of cells. Their receptors are located on the outside of the plasma membrane in order for them to be able to convey a message without entering cells. Insulin binds to the membrane-spanning insulin receptor on the surface of target cells, and initiates a signal cascade that results in an increase in the number of glucose transporters at the cell membrane, and a subsequent increase in glucose uptake.

### G proteins and second messengers

**Figure 12.**
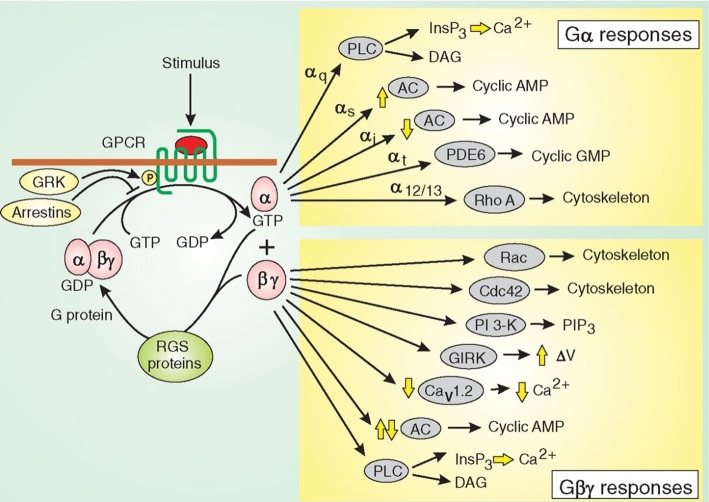
GPCR and heterotrimeric G-protein signalling. The ligand bound to the GPCR is shown in red. Binding allows the exchange of GDP for GTP by the associated G protein, and dissociation of the protein into Gα and Gβγ subunits. These then have downstream effects on a range of proteins, thereby propagating the signal from the bound ligand. Yellow arrows indicate either activation (up arrow) or inhibition (down arrow) of the targets. Regulators of G-protein signalling (RGS) proteins aid the GTPase activity of the G protein to turn off the signal. Arrestin can bind the receptor following GPCR phosphorylation by G-protein receptor kinase (GRK), desensitizing the receptor to further signalling. Reproduced from Berridge, M.J. (2012) Cell Signalling Biology; doi:10.1042/csb0001002, with permission.

Many cell-surface receptors share structural features, including seven membrane-spanning helices. These 7TM receptors bind their ligand (the messenger molecule) on the extracellular side of the membrane, and bind a GTP-binding protein (G protein) on the intracellular side. Due to this interaction with G proteins, these receptors are called G-protein-coupled receptors (GPCRs). When the ligand binds the GPCR, the receptor undergoes conformational changes that are transferred through the membrane-spanning region to the bound G protein. This change in structure allows the G protein to exchange a bound GDP molecule for a GTP molecule, and thereby switch from an inactive state to an active state. G proteins consist of three subunits—α, β and γ. An inactive, GDP-bound G protein consists of all three subunits, with the nucleotide bound in the α subunit. When the GPCR binds the ligand, the G protein is activated and the α subunit, now with GTP bound to it, dissociates from the complex (Figure 12). This activated α subunit now has an exposed face (where the β and γ subunits were bound) and can bind proteins to propagate the signal. An example of this downstream signalling from GPCRs is the activation of adenylate cyclase by the GTP-bound α subunit in the case of the β-adrenergic receptor when it binds its ligand, adrenaline (epinephrine). The effect of this adenylate cyclase activation is an increase in cAMP production from ATP, leading to downstream effects. The dissociated βγ dimer also has downstream effects. The α subunit has GTPase activity so that it can convert the bound GTP back to GDP. The GDP-bound subunit then returns to and binds the β and γ subunits ready for another cycle of signalling.

After the initial ligand interaction with the GPCR and the G-protein dissociation, the message is then carried by second messengers activated by the signal cascade. In the example that has just been given, the G protein associated with the β-adrenergic receptor activates adenylate cyclase, increasing the production of cAMP, which is a widely used second messenger. Most of the effects of cAMP are due to the activation of protein kinase A (PKA). PKA phosphorylates target enzymes to modify their activities. In the case of adrenaline, PKA activates enzymes involved in the production of glucose from glycogen stores, and inhibits enzymes involved in the production of more glycogen.

**Figure 13.**
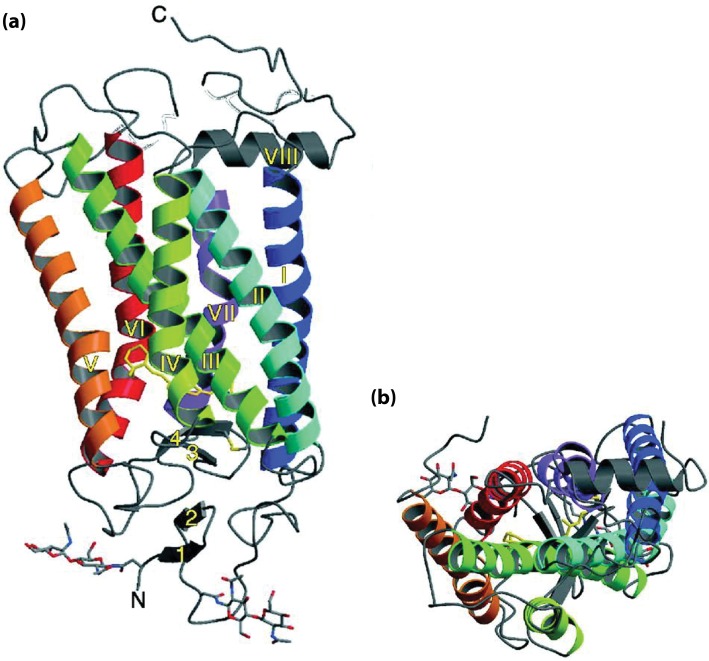
The crystal structure of rhodopsin. A ribbon representation of the first crystal structure of rhodopsin is shown in the plane of the membrane (**a**) and from the cytoplasmic side (b). The N- and C-termini are labelled, as are the seven transmembrane helices (I-VII). Adapted from Figure 2 from Palczewski, K., Kumasaka, T., Hori, T., Behnke, C.A., Motoshima, H., Fox, B.A., Le Trong, I., Teller, D.C., Okada, T., Stenkamp, R.E. et al. (2000) Crystal structure of rhodopsin: a G-protein-coupled receptor. Science **289**, 739–745.

Around 25% of drugs are targeted at GPCRs, so an understanding of their structures and functions is crucial in the fight against disease. As explained earlier, membrane proteins are notoriously difficult to crystallize due to their hydrophobic nature, and GPCRs have a very small hydrophilic area. Some techniques, such as the production of an antibody–receptor complex to increase hydrophilicity, have been successful in aiding crystallization. Rhodopsin (Figure 13) was crystallized in 2000, followed by the related β_2_-adrenergic receptor in 2007. Since then, several more GPCR structures have been solved, providing valuable information that can help computational biologists to work out the detailed mechanisms of GPCR signalling. Molecular dynamics simulations have been performed on the interactions between GPCRs and their partner G proteins using the crystal structures available to inform the modelling process. These studies will play an important part in helping us to understand how the helices in the GPCRs move and twist in order to convey the extracellular signal to the intracellular G protein.

### Nerve impulses

Nerve impulses are able to occur because biological membranes are impermeable to ions, so a membrane potential can be generated across them, with more of one charge on one side than on the other. These membrane potentials are generated and altered by ion channels. A nerve impulse, or action potential, is generated when a membrane is depolarized upon influx of positively charged ions into the cell. The resting potential in a neuron is around –70 mV, maintained by K^+^ channels and the Na^+^/K^+^-ATPase. When an action potential is generated, voltage-dependent Na^+^ channels open once the cell membrane has crossed the threshold potential of around –60 mV. This allows a fast influx of Na^+^ down its electrochemical gradient, increasing the membrane potential (i.e. reducing its negative value). This influx of positive charges enables the inside of the cell to become positively charged compared with the extracellular environment, as the membrane potential exceeds 0 mV. The depolarization itself inhibits the Na^+^ channels, so no more ions enter the cell. To restore the negative resting potential, voltage-dependent K^+^ channels open, allowing K^+^ ions to move out of the cell, thus making the inside of the cell more negative. An after-potential (hyperpolarization) can then occur, whereby the membrane potential decreases below –70 mV before being restored by the action of ion channels and ATPases.

## Membranes in health and disease

We have seen how membranes, and the membrane proteins within them, function in healthy cells and organisms. We shall now consider what happens in disease, and how we can use our knowledge of membrane proteins to make new drugs to treat disease.

### Serious disease results from non-functional ion channels

Cystic fibrosis is an autosomal recessive disease that results from mutations in the *CFTR* gene. This gene encodes a Cl^−^ channel that has a vital role in regulating the viscosity of mucus on membranes such as those in the lungs. In healthy individuals, transport of Cl^−^ ions out of the cells through CFTR is followed by water, and mucus of the right viscosity is produced. However, lack of Cl^−^ channels results in thick, dehydrated mucus, and consequently cystic fibrosis patients have difficulty in breathing and a predisposition to chest infections. Most cases of cystic fibrosis are caused by the ΔF508 mutation, which is a deletion of a phenylalanine residue at position 508 in the protein. Like nearly all membrane proteins, CFTR is translated on ribosomes at the ER and then moves through the secretory pathway to the plasma membrane, where it carries out its transport role. The single amino acid deletion of F508 causes the protein to misfold, and instead of moving out to the plasma membrane, it is held in the ER by the protein quality control machinery. Therefore very few CFTR molecules reach the plasma membrane in people with the ΔF508 mutation, and this results in serious disease.

Diseases such as this are not easily treated. Blocking the protein quality control machinery is not an option, because it would lead to the release of other misfolded proteins, with potentially disastrous consequences. Although some current drug treatments can ameliorate the symptoms of the disease, it is hoped that gene therapy might become routine as it addresses the cause of the problem. Treatment of patients with an artificial, functional version of the gene enables them to produce a working CFTR protein that can be expressed at the plasma membrane. Although this is not a complete cure, it is a potentially effective way to greatly reduce the symptoms of cystic fibrosis in the lungs. As DNA is a large hydrophilic molecule, it cannot be simply administered like many other drugs. Delivery of gene therapy is a challenge, and this is one reason why it is difficult to treat patients in this way, but methods of delivering new genetic material into cells have been developed. Viruses can be used to deliver the *CFTR* gene to cells, by harnessing their ability to inject cells with foreign DNA or RNA. Patients may also be able to be given liposomes containing the functional gene, which fuse with cell membranes and deliver the therapeutic gene. Gene therapy is a growing and important area of research, and it is hoped that many diseases, including some cancers, will eventually be able to be treated using DNA.

### Membrane proteins provide an entry point for viruses

Viruses that attack the human body can use the body's own membrane proteins to recognize their target cells. HIV attacks cells of the immune system. A protein on the surface of HIV called gp120 binds to CD4 protein molecules on the surface of T-cells that are involved in immunoregulation, and allows fusion of the virus with the host cell. Once the contents of the virus have entered the CD4-positive cell, the HIV genome is integrated with the host genome and uses the host machinery to make new copies of the virus. Over time, the numbers of CD4 T-cells are reduced by the virus, and the patient's immune system eventually becomes so compromised that they are unable to fight invading pathogens. Many therapeutic agents have been created to help to fight HIV, and the interaction between CD4 and gp120 is just one of the points at which drugs can be used to stop the progression of the virus.

### Toxins use endocytosis to gain entry to cells and block neurotransmission

**Figure 14.**
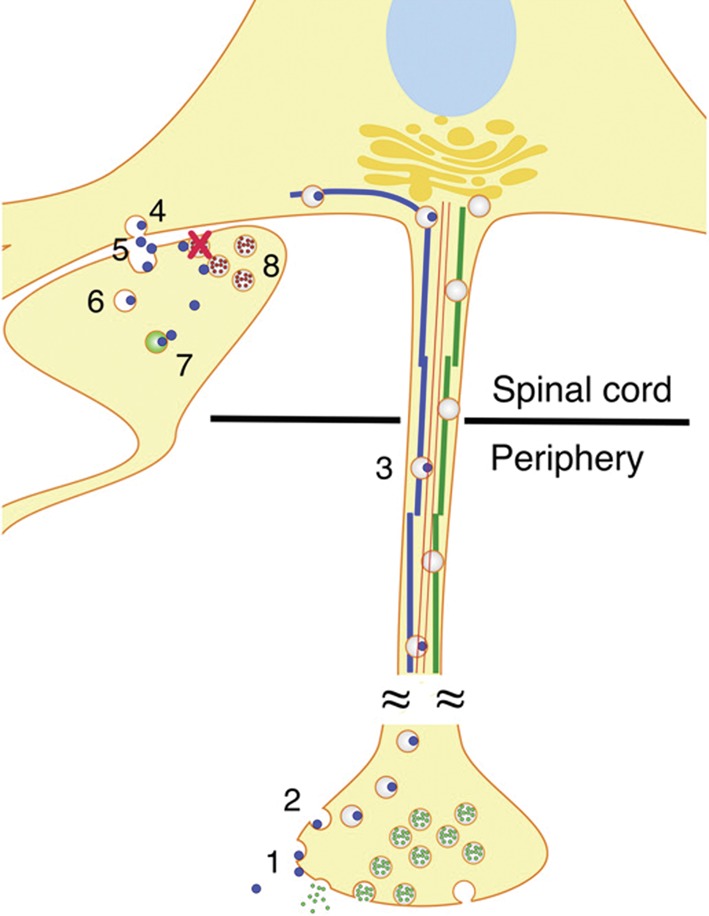
Mechanism of action of tetanus toxin. Tetanus toxin (blue circles) enters the presynaptic membranes of motor neurons by endocytosis, and moves up the axon to the dendrites that connect the motor neuron to an inhibitory interneuron. Microtubules (blue and green lines) and actin filaments (red lines) allow retrograde transport of the toxin. TeNT acts on the inhibitory interneuron, where it prevents the release of glycine (red dots), shown by a red cross. Small green dots represent neurotransmitter both inside and being released from vesicles. Adapted from Figure 2 from Rossetto, O., Scorzeto, M., Megighian, A. and Montecucco, C. (2013) Tetanus neurotoxin. Toxicon **66**, 59–63.

Various toxins interfere with the transmission of messages across biological membranes. Tetanus neurotoxin (TeNT) and botulinum neurotoxin (BoNT) are both protein toxins that affect nerve impulse transmission between nerves and muscles. TeNT is produced by a soil bacterium and causes the skeletal muscle spasms that characterize tetanus infection. TeNT-producing bacteria generally enter the body through wounds, and TeNT binds glycolipids enriched at presynaptic membranes of motor neurons (Figure 14). TeNT then undergoes endocytosis and moves up the axon to the dendrites that connect the motor neuron to an inhibitory interneuron. TeNT is released into the synapse between these two cells and is endocytosed into the inhibitory interneuron. Acidification of vesicles containing TeNT causes the protein toxin to break apart into two domains. One of these, the L domain, is translocated into the cytoplasm of the interneuron, where it uses its proteolytic activity to cleave vesicle-associated membrane protein (VAMP). Under normal circumstances, VAMP is part of the protein complex that allows synaptic vesicles to fuse with the presynaptic membrane and release inhibitory neurotransmitters. The action of the L-domain protease of TeNT means that VAMP can no longer function, inhibiting neurotransmitter release across the synapse. As this occurs in inhibitory interneurons, the resulting effect is prolonged skeletal muscle contraction, as no inhibition is conveyed to the motor neuron to allow relaxation.

Botulinum neurotoxin acts in a similar way to TeNT, but has the opposite effect. Like TeNT, it is released by bacteria. BoNT binds to and is internalized by the presynaptic membrane of motor neurons at the neuromuscular junction. It is released from endocytic vesicles into the cytoplasm of the motor neuron, where it acts on the SNARE complex to inhibit the fusion of synaptic vesicles and release of excitatory neurotransmitters at the neuromuscular junction. This has the effect of blocking muscle contraction and causing paralysis in people infected with botulism. Despite its sometimes lethally toxic nature, BoNT is increasingly used by people who wish to look younger. The toxin is injected into the muscles of the face to cause paralysis, thereby reducing wrinkles and lines in the skin. When used in this way it is referred to as Botox.

### Membrane proteins are the target of many drugs

Membrane proteins are important drug targets. As our structural and functional knowledge of membrane proteins expands, it is becoming possible to design more effective medicines. Computational tools are becoming an increasingly important part of the process. One important class of drug targets are pore-forming membrane proteins encoded by viruses. HIV, influenza and polio, among other viruses, encode membrane proteins that form pores in the host cell membranes in order to cause leakage and promote infection. One of these pore-forming proteins was formerly used as a drug target in the treatment of influenza. NMR studies have provided structural information about the pore-forming Vpu protein from HIV-1. Using these data together with structural information about pores with similar sequences, computational models of the structure of the channel in the host membrane can be produced. These models, combined with advanced biophysical techniques, are invaluable for predicting sites for potential drug molecule binding which can then be tested both computationally and experimentally.

More drugs target GPCRs than any other single group of proteins. As explained earlier, conformational changes in the GPCRs permit signals to cross the membrane. However, these large changes in conformation give the proteins flexibility, which makes it difficult for researchers to pinpoint the structures of the GPCRs in any one conformation. Solving the structures of different conformations using X-ray crystallography is a challenging task. As GPCRs are such important drug targets, much research has been focused on solving their structures in order to inform the discovery of new drugs. Computational methods have again proved crucial to understanding the detailed molecular structure of these proteins. Molecular dynamics simulations have been used to aid our understanding of the molecular changes that occur during GPCR activation, and also which lipids are required for the GPCR to function. Although molecular dynamics simulations are a key technique in this area of research, they have some limitations. The biological membranes that surround cells are extremely complex and contain different types of lipids and proteins, both within and associated with the membrane. At present, the time and financial resources required to provide the computational power to simulate such a complex environment are often prohibitive. Simpler models are therefore produced which, although they are able to predict conformational changes in receptors such as GPCRs, may omit other interactions that are important in the activity of membrane proteins. As is often the case, a combination of techniques will be required to gain greater insight into the conformational flexibility of the GPCRs. It is apparent from research conducted to date that these receptors can adopt many different conformations, rather than having just a simple ‘on’ and ‘off’ mechanism. Using different drug molecules to stabilize different conformations in different signalling pathways may be the best approach to finding more effective medicines in the future. There is now much pressure on researchers to replace, refine and reduce the use of animals in drug discovery (an approach referred to as the ‘three Rs’). By using computers in the early stages of the process to model drug–target interactions, researchers can produce much more promising compounds to test in experiments and drug trials.

## Closing remarks

Biological membranes allow life to exist. From simple unicellular prokaryotes to complex multicellular eukaryotes such as humans, the properties of the membranes that surround cells are remarkably similar. Our understanding of the structure of these lipid bilayers is now expanding rapidly as a result of significant advances in biophysical techniques and the huge computational power now available to researchers. The proteins that inhabit these membranes allow messages to be sent and received so that the cell can communicate with the external environment. Many messages are relayed by hydrophilic molecules that require receptors to transmit information across the bilayer. It is this step that is targeted by the majority of drugs which are on the market today, as it enables us to modify the message before it enters the cell. An understanding of how membrane proteins work, how they reach the correct destinations and how we can alter their functions is key to the fight against human disease.

**Abbreviations**
BoNT: botulinum neurotoxin
CFTR: cystic fibrosis transmembrane conductance regulator
COP: coat protein
ER: endoplasmic reticulum
ERGIC: ER–Golgi intermediate compartment
ESCRT: endosomal sorting complex required for transport
GFP: green fluorescent protein
GPCR: G-protein-coupled receptor
GPI: glycosyl-phosphatidylinositol
G protein: GTP-binding protein
NMR: nuclear magnetic resonance
PC: phosphatidylcholine
PDB: Protein Data Bank
PE: phosphatidylethanolamine
PKA: protein kinase A
PS: phosphatidylserine
SERCA: sarco/endoplasmic reticulum Ca^2+^-ATPase
SR: sarcoplasmic reticulum
SRP: signal recognition particle
TeNT: tetanus neurotoxin
VAMP: vesicle-associated membrane protein
